# Supplementary material for: WASH interventions and child diarrhea at the interface of climate and socioeconomic position in Bangladesh
Source: Nat Commun. 2024 Feb 20;15:1556. doi: 10.1038/s41467-024-45624-1 (PMC10879131; doi:10.1038/s41467-024-45624-1)
Supplement: Supplementary file 1 — Supplementary Information [file 41467_2024_45624_MOESM1_ESM.pdf]

Supplementary Material to

WASH interventions and child diarrhea at the interface of climate and socioeconomic position in Bangladesh

Pearl Anne Ante-Testard*, Francois Rerolle, Anna T. Nguyen, Sania Ashraf, Sarker Masud Parvez, Abu Mohammed Naser, Tarik Benmarhnia, Mahbubur Rahman, Stephen P. Luby, Jade Benjamin-Chung, Benjamin F. Arnold

***Corresponding author:** Pearl Anne Ante-Testard; Francis I. Proctor Foundation and Department of Ophthalmology, University of California, San Francisco, CA, United States; pearl.ante@ucsf.edu.

Supplementary Table 1. Summary of household characteristic means and standard deviation by wealth index quintiles. The household asset-based characteristics were included in the construction of the wealth index through a principal component analysis. The factor loading was the eigenvector from the first principal component.

Asset-based characteristics included in the wealth index	Wealth Index Quintiles					Factor Loading
	1 (N=1110)	2 (N=1110)	3 (N=1110)	4 (N=1109)	5 (N=1112)	
	Mean (SD)	Mean (SD)	Mean (SD)	Mean (SD)	Mean (SD)	
**Land owned in acres (Mean, SD)**	0.07 (0.07)	0.10 (0.10)	0.13 (0.17)	0.17 (0.33)	0.26 (0.34)	0.09
**Improved wall material (wood, brick, thin)**	0.64 (0.48)	0.66 (0.48)	0.79 (0.41)	0.77 (0.42)	0.74 (0.44)	0.06
**Improved floor material (wood, concrete)**	0.003 (0.052)	0.01 (0.11)	0.05 (0.21)	0.09 (0.28)	0.38 (0.49)	0.17
**Household has electricity**	0.14 (0.35)	0.35 (0.48)	0.52 (0.50)	0.93 (0.25)	1.0 (0.07)	0.42
**Household has refrigerator**	0.00 (0.00)	0.001 (0.030)	0.003 (0.052)	0.03 (0.17)	0.36 (0.48)	0.15
**Household has bicycle**	0.08 (0.27)	0.22 (0.41)	0.34 (0.47)	0.40 (0.49)	0.51 (0.50)	0.21
**Household has motorcycle**	0.002 (0.042)	0.004 (0.060)	0.03 (0.16)	0.06 (0.24)	0.23 (0.42)	0.11
**Household has sewing machine**	0.01 (0.09)	0.03 (0.18)	0.05 (0.21)	0.08 (0.27)	0.16 (0.36)	0.07
**Has black and white or colored TV**	0.002 (0.042)	0.04 (0.20)	0.12 (0.33)	0.39 (0.49)	0.94 (0.25)	0.41
**Has one or more wardrobe**	0.01 (0.09)	0.05 (0.22)	0.09 (0.28)	0.16 (0.37)	0.52 (0.50)	0.22
**Has one or more table**	0.22 (0.42)	0.66 (0.48)	0.85 (0.36)	0.92 (0.28)	0.98 (0.14)	0.34
**Has one or more chair**	0.19 (0.39)	0.65 (0.48)	0.87 (0.34)	0.94 (0.24)	1.0 (0.07)	0.36
**Has one or more khat (type of bed)**	0.07 (0.25)	0.37 (0.48)	0.71 (0.46)	0.91 (0.29)	1.0 (0.07)	0.44
**Has one or more chouki (type of chair)**	0.95 (0.22)	0.83 (0.38)	0.77 (0.42)	0.77 (0.42)	0.63 (0.48)	-0.14
**Has one or more mobile**	0.56 (0.50)	0.82 (0.38)	0.93 (0.26)	0.98 (0.16)	0.99 (0.09)	0.19

SD=standard deviation; N=total number.

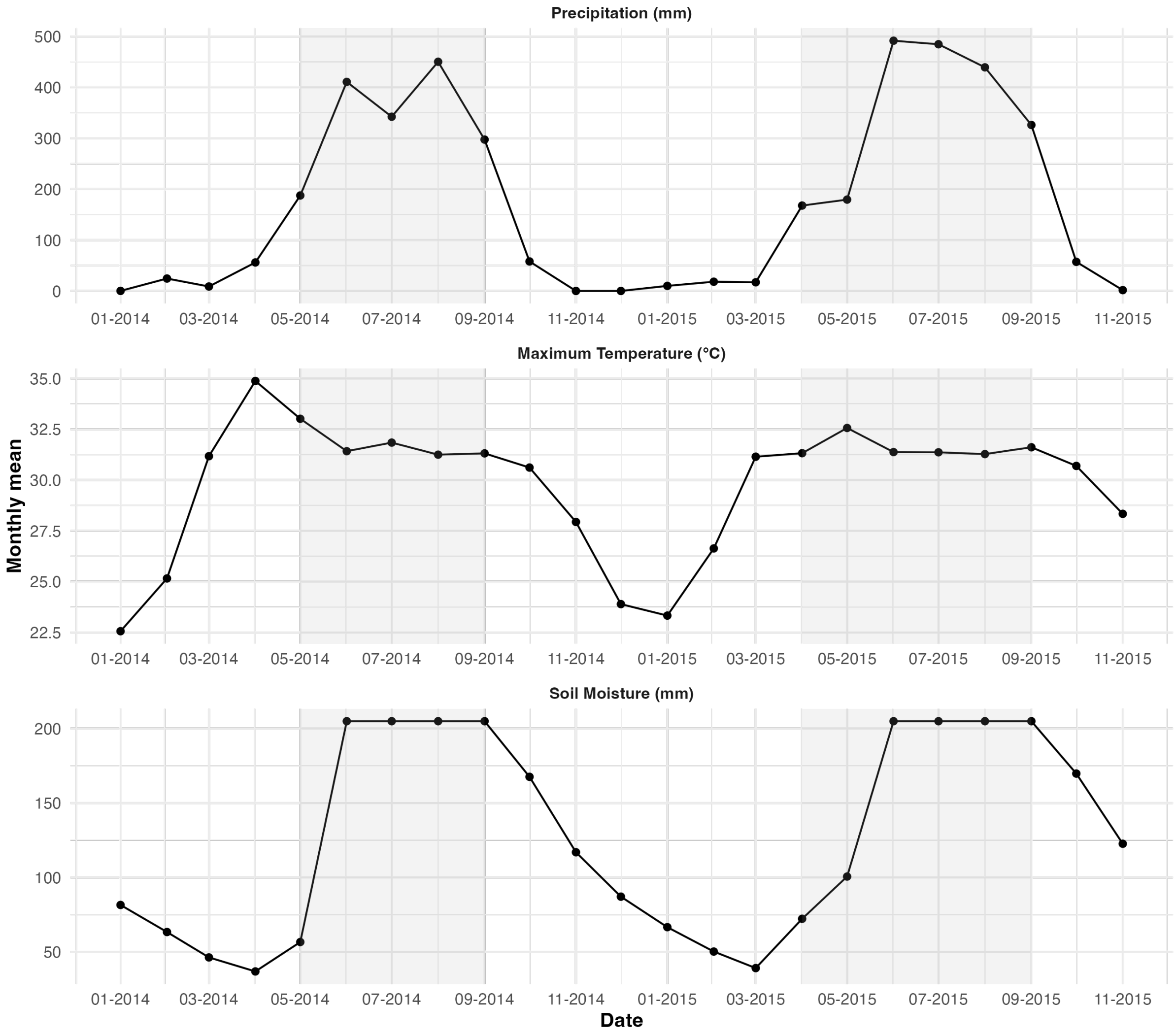

Supplementary Fig. 1: Climate characteristics during the study. Monthly mean by precipitation, maximum temperature and soil moisture during the study trial. Shaded areas illustrate the monsoon season defined as weeks with elevated precipitation (May 27 – September 27, 2014, and April 1 – September 26, 2015).

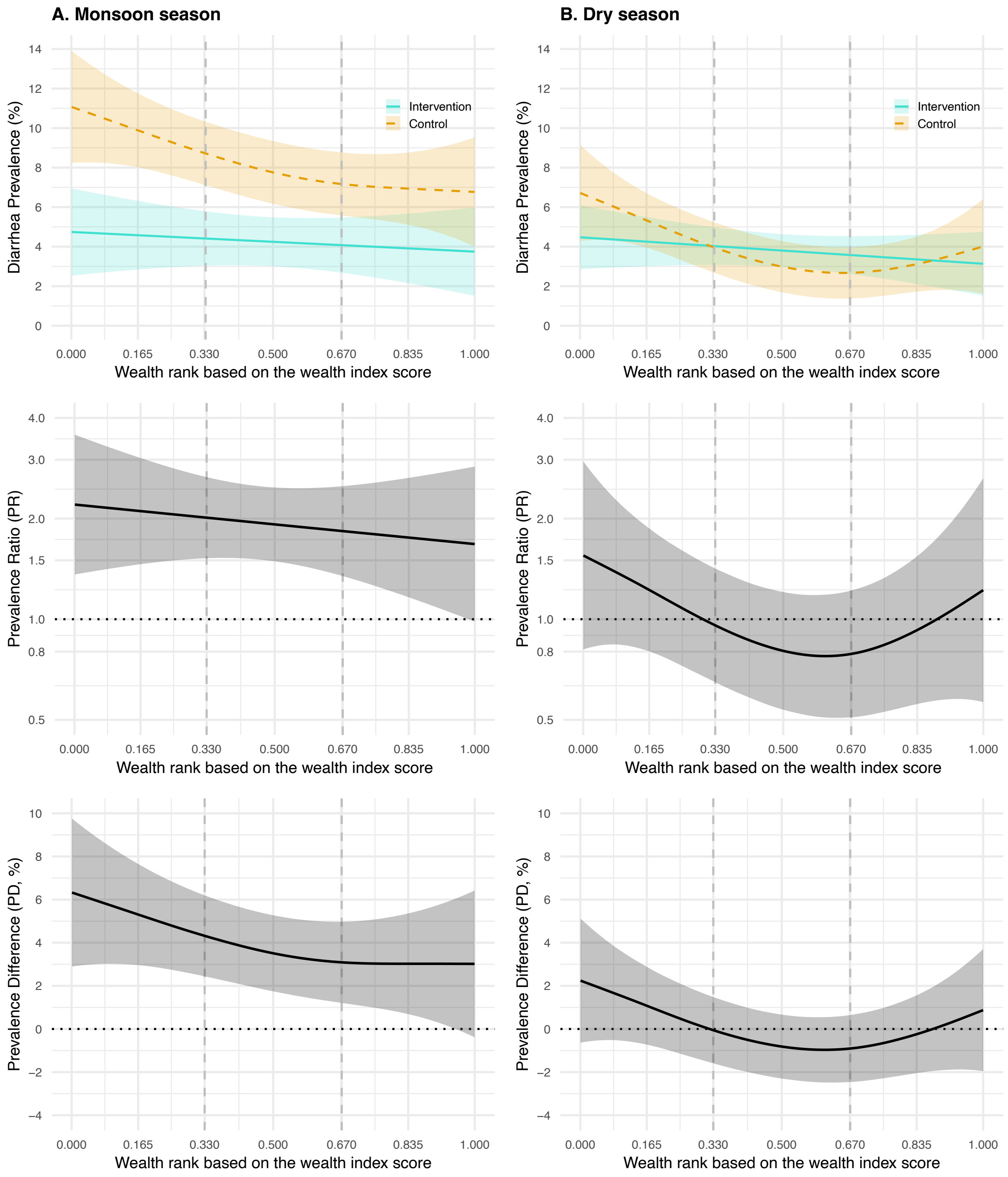

Supplementary Fig. 2: Effect of WASH interventions by socioeconomic position using a continuous wealth score. Left panels show estimates during the monsoon season (**A**). Right panels show estimates during the dry season (**B**). The Y-axes for the prevalence ratios are on a log scale. The central lines represent the central estimates. Shaded areas represent 95% confidence intervals. The vertical dashed lines are the cut-offs for the wealth tertiles. Please refer to Table 2 for the sample size (**A:** n=3,981, **B:** n=4459).

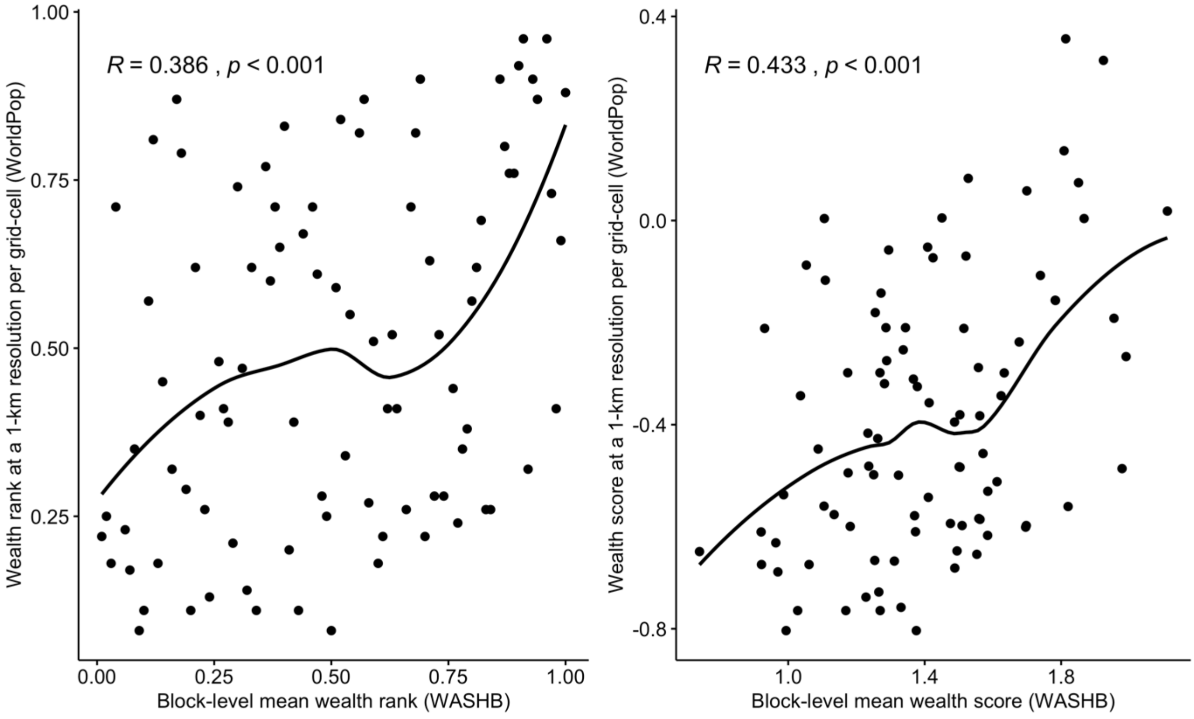

Supplementary Fig. 3. Wealth rank and wealth scores based on the WorldPop predicted wealth index per grid-cell compared to block-level mean wealth rank based on the wealth index and block-level mean wealth scores measured from the WASH Benefits Bangladesh trial. The correlation was evaluated using the Spearman correlation coefficient. The fitted line was generated using a loess function.

Supplementary Table 2. Summary of baseline characteristics by maternal education.

Baseline characteristics	Maternal Education		
	No education (N=259)	Primary (1-5y) (N=591)	Secondary (>5y) (N=758)
Asset-based characteristics included in the wealth index			
Land owned in acres (Mean, SD)	0.0723 (0.0742)	0.0946 (0.118)	0.160 (0.212)
Improved wall material (wood, brick, thin)	172 (66.4%)	468 (79.2%)	541 (71.4%)
Improved floor material (wood, concrete)	4 (1.5%)	31 (5.2%)	106 (14.0%)
Household has electricity	99 (38.2%)	287 (48.6%)	509 (67.2%)
Household has refrigerator	0 (0%)	8 (1.4%)	100 (13.2%)
Household has bicycle	52 (20.1%)	109 (18.4%)	279 (36.8%)
Household has motorcycle	0 (0%)	18 (3.0%)	81 (10.7%)
Household has sewing machine	0 (0%)	11 (1.9%)	75 (9.9%)
Has black and white or colored TV	15 (5.8%)	98 (16.6%)	268 (35.4%)
Has one or more wardrobe	12 (4.6%)	48 (8.1%)	171 (22.6%)
Has one or more table	140 (54.1%)	394 (66.7%)	600 (79.2%)
Has one or more chair	139 (53.7%)	372 (62.9%)	625 (82.5%)
Has one or more khat (type of bed)	89 (34.4%)	309 (52.3%)	556 (73.4%)
Has one or more chouki (type of chair)	228 (88.0%)	455 (77.0%)	568 (74.9%)
Has one or more mobile	179 (69.1%)	479 (81.0%)	680 (89.7%)
Asset-based characteristics not included in the wealth index			
Primary water source: shallow tubewell	179 (69.1%)	447 (75.6%)	557 (73.5%)
Store drinking water	136 (52.5%)	295 (49.9%)	331 (43.7%)
Reported treating water today/tomorrow	0 (0%)	2 (0.3%)	2 (0.3%)
Own their latrine	91 (35.1%)	214 (36.2%)	371 (48.9%)
Latrine has concrete slab	203 (78.4%)	504 (85.3%)	708 (93.4%)
Latrine has functional water seal	31 (12.0%)	81 (13.7%)	212 (28.0%)
No visible feces on floor of latrine	88 (34.0%)	193 (32.7%)	361 (47.6%)
Has a potty for defecation	3 (1.2%)	26 (4.4%)	85 (11.2%)
Primary handwashing location has water/soap	19 (7.3%)	81 (13.7%)	209 (27.6%)
Household has radio	6 (2.3%)	13 (2.2%)	44 (5.8%)
Has one or more clock	39 (15.1%)	133 (22.5%)	352 (46.4%)

SD=standard deviation; N=total number.

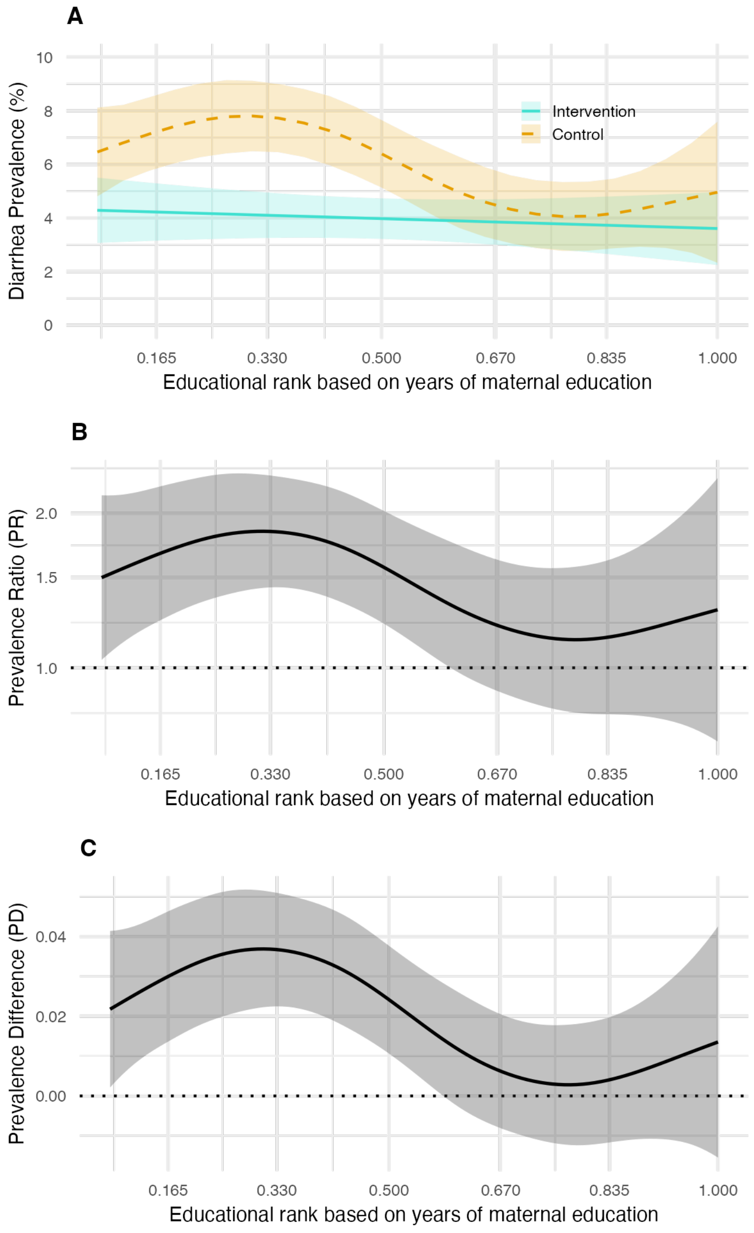

Supplementary Fig. 4: Effect of WASH interventions on diarrhea by mother's educational rank based on the number of years of education. A: Diarrhea prevalence along the continuous educational rank in the control and intervention groups. The central estimates as depicted by splines represent the diarrhea prevalence. Shaded area represents 95% confidence intervals. **B:** Prevalence ratio of child diarrhea along the continuous educational rank in the control and intervention groups. The central estimates as depicted by splines represent the prevalence ratios. Shaded area represents 95% confidence intervals. **C:** Prevalence difference of child diarrhea along the continuous educational rank in the control and intervention groups. The central estimates as depicted by splines represent the prevalence differences. Shaded area represents 95% confidence intervals. **A, B and C:** Please refer to Table 2 for the sample size (n=8,440).

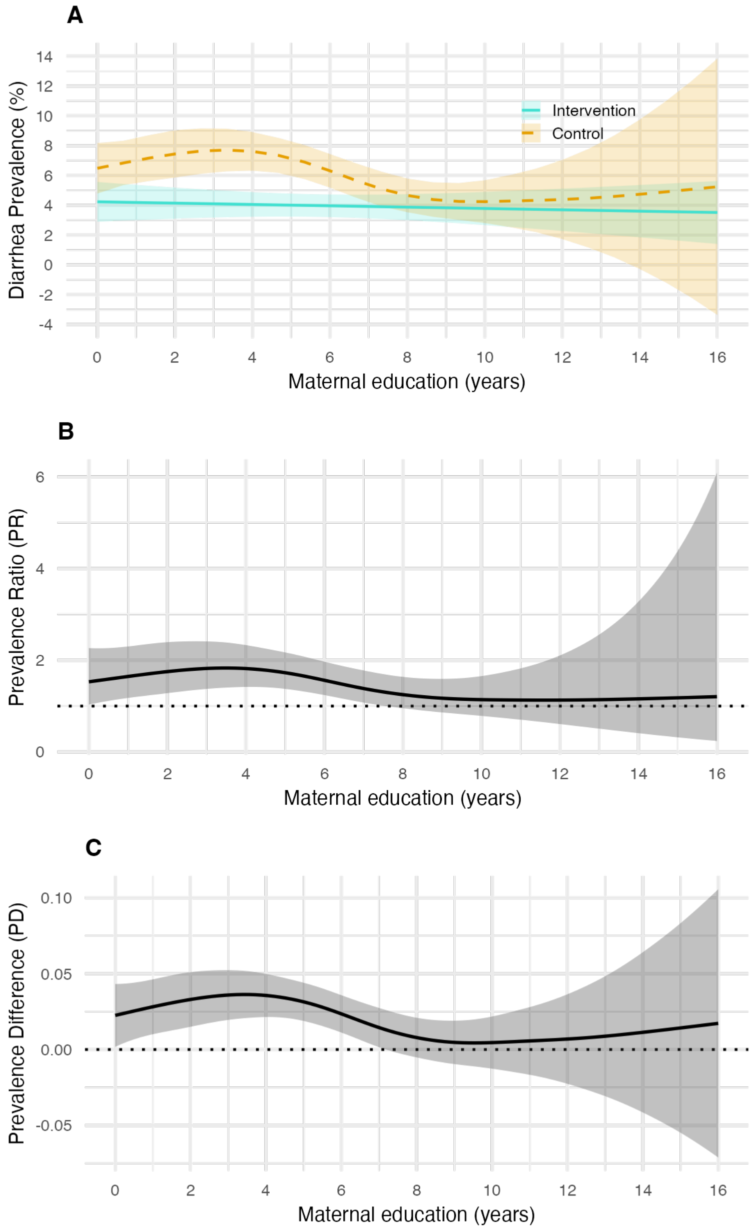

Supplementary Fig. 5: Effect of WASH interventions on diarrhea by mother's educational years completed. A: Diarrhea prevalence across years of completed education in the control and intervention groups. The central estimates as depicted by splines represent the diarrhea prevalence. Shaded area represents 95% confidence intervals. **B:** Prevalence ratio of child diarrhea across years of completed education in the control and intervention groups. The central estimates as depicted by splines represent the prevalence ratios. Shaded area represents 95% confidence intervals. **C:** Prevalence difference of child diarrhea across years of completed education in the control and intervention groups. The central estimates as depicted by splines represent the prevalence differences. Shaded area represents 95% confidence intervals. **A, B and C:** Please refer to Table 2 for the sample size (n=8,440).

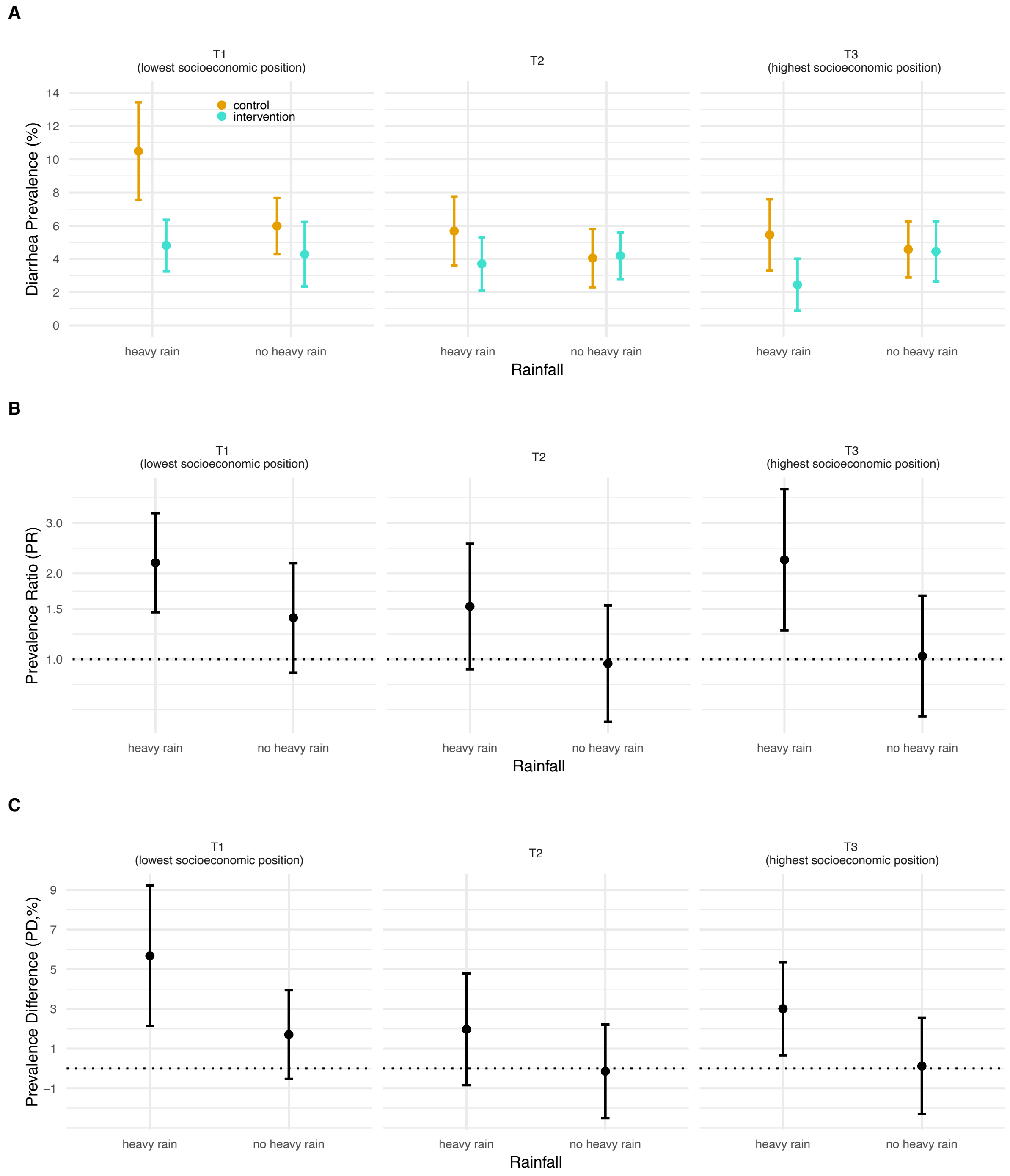

Supplementary Fig. 6: Effect of WASH interventions by socioeconomic position and rainfall. A: Diarrhea prevalence along the tertiles of wealth index in the event of heavy rain versus no heavy rain in the control and intervention groups. The central estimates as depicted by circles represent the diarrhea prevalence. Error bars represent 95% confidence intervals. **B:** Prevalence ratio of child diarrhea along the tertiles of wealth index in the event of heavy rain versus no heavy rain in the control and intervention groups. Y-axis is on a log scale**.** The central estimates as depicted by circles represent the prevalence ratios. Error bars represent 95% confidence intervals. **C:** Prevalence difference of child diarrhea along the tertiles of wealth index in the event of heavy rain versus no heavy rain in the control and intervention groups. The central estimates as depicted by circles represent the prevalence differences. Error bars represent 95% confidence intervals. **A, B and C:** Please refer to Table 2 for the sample size (n=8,440).

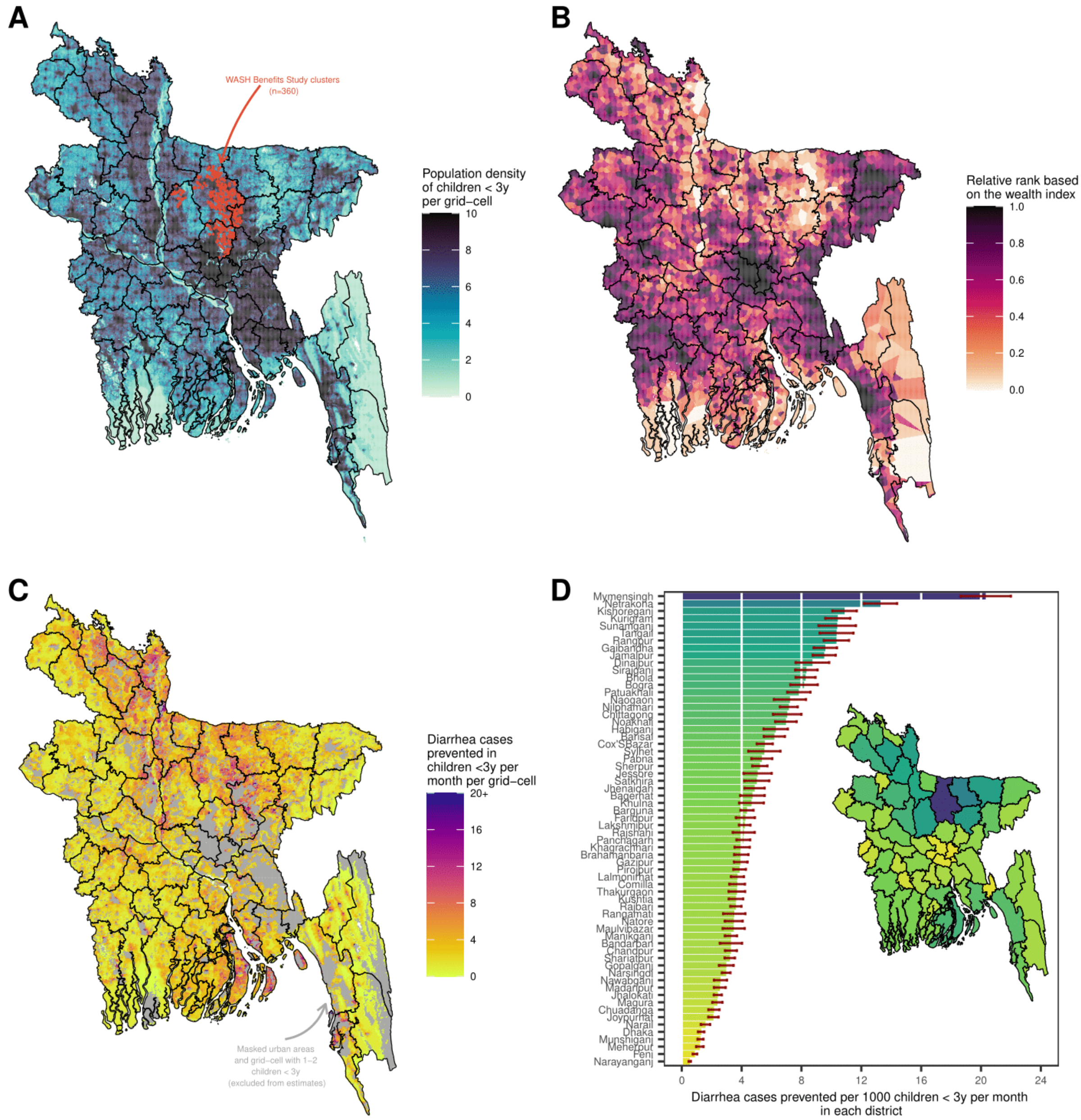

Supplementary Fig. 7: Diarrhea cases prevented by a combined WASH intervention along a continuous wealth gradient per grid-cell averaged over the entire year at a 1-kilometer resolution and per district in rural Bangladesh. A: WASH Benefits Study clusters that were included in the analysis (n=360) and gridded population density of children <3 years estimated from WorldPop. **B:** Distribution of relative wealth rank based on the Demographic and Health Surveys wealth index with Bayesian model-based geostatistics and high resolution gridded spatial covariates and aggregated phone data per grid-cell at 1-km resolution estimated from WorldPop. **C:** The projected diarrhea cases prevented by combined WASH per month by wealth per grid-cell at 1-km resolution. Masked urban areas based on the Global Human Settlement and grid-cell with only 1-2 children < 3 years. **D:** The projected diarrhea cases prevented by combined WASH per 1000 children under 3 years per month in panel C aggregated for each administrative district (n=64 districts). The bars represent the sum of the grid-cell projected diarrhea cases prevented by combined WASH averaged over the entire year in children < 3 years per month at the district level. Error bars represent 95% confidence intervals.

Supplementary Text 1. We assessed the effect modification of WASH by socioeconomic position, season and jointly by socioeconomic position and season. We assessed the effect modification by comparing the models with and without the interaction term through a Wald-type F test to test for statistical significance.

Effect modification by socioeconomic position

$$P(diarrhea) \sim f(WASH, SEP, SEP{:}WASH)$$

versus

$$P(diarrhea) \sim f(WASH, SEP)$$

Effect modification by season

$$P(diarrhea) \sim f(WASH, season, season{:}WASH)$$

versus

$$P(diarrhea) \sim f(WASH, season)$$

Joint effect modification by socioeconomic position and season

$$P(diarrhea) \sim f(WASH, SEP, season, SEP{:}WASH, season{:}WASH,$$
$$SEP{:}season{:}WASH)$$

versus

$$P(diarrhea) \sim f(WASH, SEP, season),$$

where SEP = socioeconomic position, WASH = Water, Sanitation and Handwashing.
