# Supplementary material for: WASH interventions and child diarrhea at the interface of climate and socioeconomic position in Bangladesh
Source: Nat Commun. 2024 Feb 20;15:1556. doi: 10.1038/s41467-024-45624-1 (PMC10879131; doi:10.1038/s41467-024-45624-1)
Supplement: Supplementary file 3 — Reporting Summary [file 41467_2024_45624_MOESM3_ESM.pdf]

nature portfolio

Corresponding author(s): Pearl Anne Ante-Testard

Last updated by author(s): Jan 22, 2024

Reporting Summary

Nature Portfolio wishes to improve the reproducibility of the work that we publish. This form provides structure for consistency and transparency in reporting. For further information on Nature Portfolio policies, see our Editorial Policies and the Editorial Policy Checklist.

Statistics

For all statistical analyses, confirm that the following items are present in the figure legend, table legend, main text, or Methods section.

n/a	Confirmed	
☐	☒	The exact sample size (*n*) for each experimental group/condition, given as a discrete number and unit of measurement
☐	☒	A statement on whether measurements were taken from distinct samples or whether the same sample was measured repeatedly
☐	☒	The statistical test(s) used AND whether they are one- or two-sided<br>*Only common tests should be described solely by name; describe more complex techniques in the Methods section.*
☐	☒	A description of all covariates tested
☒	☐	A description of any assumptions or corrections, such as tests of normality and adjustment for multiple comparisons
☐	☒	A full description of the statistical parameters including central tendency (e.g. means) or other basic estimates (e.g. regression coefficient) AND variation (e.g. standard deviation) or associated estimates of uncertainty (e.g. confidence intervals)
☐	☒	For null hypothesis testing, the test statistic (e.g. *F*, *t*, *r*) with confidence intervals, effect sizes, degrees of freedom and *P* value noted<br>*Give P values as exact values whenever suitable.*
☒	☐	For Bayesian analysis, information on the choice of priors and Markov chain Monte Carlo settings
☒	☐	For hierarchical and complex designs, identification of the appropriate level for tests and full reporting of outcomes
☐	☒	Estimates of effect sizes (e.g. Cohen's *d*, Pearson's *r*), indicating how they were calculated

Our web collection on statistics for biologists contains articles on many of the points above.

Software and code

Policy information about availability of computer code

Data collection	There was no data collection software used in this pre-specified secondary analysis of a cluster randomized trial in Bangladesh. Primary data were collected on handheld tablets using a custom study application programmed in Open Data Kit (ODK).
Data analysis	Analyses were conducted in R version 4.2.1. ("Funny Looking Kid"). Analysis scripts and instructions to reproduce all analyses are available in the Open Science Framework repository (https://osf.io/xwndg/).

For manuscripts utilizing custom algorithms or software that are central to the research but not yet described in published literature, software must be made available to editors and reviewers. We strongly encourage code deposition in a community repository (e.g. GitHub). See the Nature Portfolio guidelines for submitting code & software for further information.

Data

Policy information about availability of data

All manuscripts must include a data availability statement. This statement should provide the following information, where applicable:

- Accession codes, unique identifiers, or web links for publicly available datasets
- A description of any restrictions on data availability
- For clinical datasets or third party data, please ensure that the statement adheres to our policy

The pre-analysis plan, de-identified data and accessible links to the spatial and climate data are available through the Open Science Framework (https://osf.io/xwndg/).

Research involving human participants, their data, or biological material

Policy information about studies with human participants or human data. See also policy information about sex, gender (identity/presentation), and sexual orientation and race, ethnicity and racism.

Reporting on sex and gender	We conducted an overall analysis across sex and gender. WASH interventions are not currently implemented based on sex or gender at the individual level; rather at the household level.
Reporting on race, ethnicity, or other socially relevant groupings	N/A
Population characteristics	The study included children under 3 years old residing in rural communities within the districts of Gazipur, Kishoreganj, Mymesingh, and Tangail in Bangladesh. It centered on both index children within the birth cohort and other children below 3 years of age who were residing in the same compound at the time of study enrollment. Our secondary analysis focused on survey rounds 1 and 2, conducted in 2014 and 2015, respectively.
Recruitment	Enrollment began in June 2012 for the original study trial. Each study cluster included 8 eligible pregnant women. Compounds within the same cluster were situated in proximity, allowing a single facilitator to conveniently access each participant by walking. It was possible to include multiple clusters in a village, as long as these clusters were at least a 15-minute walk apart (approximately 1 km.) from each other.
Ethics oversight	The protocol of the original study was approved by the Ethical Review Committee at the International Centre for Diarrhoeal Disease Research, Bangladesh (PR-11063), the Committee for the Protection of Human Subjects at the University of California, Berkeley (2011-09-3652), the Institutional Review Board at Stanford University (25863) and at the University of California, San Francisco (22-36722). Informed consent was obtained for this study.

Note that full information on the approval of the study protocol must also be provided in the manuscript.

Field-specific reporting

Please select the one below that is the best fit for your research. If you are not sure, read the appropriate sections before making your selection.

☐ Life sciences ☒ Behavioural & social sciences ☐ Ecological, evolutionary & environmental sciences

For a reference copy of the document with all sections, see nature.com/documents/nr-reporting-summary-flat.pdf

Behavioural & social sciences study design

All studies must disclose on these points even when the disclosure is negative.

Study description	We conducted a pre-specified secondary analysis of the WASH Benefits Bangladesh cluster randomized controlled trial using quantitative methods.
Research sample	This study included 8,440 diarrhea measurements from 4,941 index children in the birth cohort, and other children living within the same compound that were younger than 3 years at the time of study enrollment from 360 clusters in rural communities in Gazipur, Kishoreganj, Mymesingh and Tangail districts in Bangladesh. We only included clusters that received the combined water, sanitation and handwashing (WSH), combined WSH + nutrition (WSH + N) and double-sized controls. Geographical matching was conducted to achieve balanced representation across locations and measurement times. We incorporated all accessible information from the original trial, with data exclusions described in the section "Data exclusions".
Sampling strategy	The study was a cluster randomized trial in rural districts of Gazipur, Kishoreganj, Mymesingh and Tangail in Bangladesh. The study recruited pregnant women identified during community-based surveys, expected to give birth within 6 months of enrollment. The trial created and randomly allocated 720 geographically matched clusters, with eight clusters per matched block, to an intervention or control group. In each block, eight clusters were randomly assigned to receive: improved water (W), improved sanitation (S), improved handwashing (H), improved nutrition (N), combined WSH, combined WSH + N, and a double-sized control arm. This analysis focused on children in control clusters and those that received the combined WSH and WSH + N interventions. Within each geographically matched block, the analysis included four clusters: 2 controls, 1 WSH, and 1 WSH + N.<br><br>The original trial's sample size calculation relied on the diarrhea outcome variable. The final design was chosen to identify a relative risk of diarrhea equal to or less than 0.7, specifically when comparing any intervention with the double-sized control arm. The control arm was enlarged twofold due to its involvement in multiple hypothesis testing. The calculations were made assuming a type I error of 0.05, a power of 0.8, a two-sided test for comparing means between two samples, and an attrition rate of 10%. The computations indicated a requirement for 90 clusters per group, each consisting of eight children.<br><br>In this secondary analysis, we computed the minimum detectable prevalence difference. This difference was estimated to be -3% percentage points when comparing the intervention and control groups by wealth tertiles (equivalent to 60 clusters per group). Additionally, for wealth tertiles during the monsoon season, the detectable difference was estimated at -5% percentage points between groups (requiring 30 clusters per group). These estimations were derived considering the actual intra-class correlation coefficient for diarrhea at the block level (calculated as 0.01) using a mixed effects model as outlined in Stoffel et al. (2017). The

	average cluster size was 23 children out of a total of 8,440 children distributed across 360 clusters. Notably, the average prevalence of diarrhea in the control group stood at 6%. These calculations were based on a two-sided type 1 error rate of 0.05 and a power level of 0.80. We show in Figure 1 of the manuscript the study participant flow from enrollment to analysis. There were 360 clusters in total with 8,440 diarrhea measurements from children aged less than 3 years old.<br><br>Details of the sample size calculation can be found in the pre-analysis plan in the Open Science Framework (https://osf.io/xwndg/).
Data collection	In this secondary analysis, we did not use data collection instruments. The original study trial used surveys and clinical measures at each follow-up. Primary data were collected on handheld tablets using a custom study application programmed in Open Data Kit (ODK). Participants and the data collectors were not masked to intervention assignment because of the nature of the interventions. Nevertheless, the data collection and intervention teams were different individuals. The results were unmasked after the completion of the primary outcome analyses.
Timing	The enrollment process started in June 2012, and evaluation of outcomes took place at survey year round 1 (2014) and survey year round 2 (2015).
Data exclusions	In this secondary analysis, we excluded children with missing outcome and children aged more than 3 years at the time of enrollment. We focused on surveys 1 and 2 (2014 and 2015, respectively, excluded baseline survey) and excluded the single WSH and nutrition arms to ensure a consistent WASH package. In total, we excluded 14,508 of 22,948 measurements.
Non-participation	Figure 1 in the manuscript summarizes the reasons for loss to follow-up of the children. However, no clusters dropped out of the trial over the two-year study period. This involved a secondary analysis of existing data, with no direct involvement of participants in the study.
Randomization	In the original study trial, randomization at block level was conducted for treatment allocation. Each of the 8 geographically matched clusters was block-randomized to the double-sized control arm or one of the six intervention arms. In a matched block, the trial randomly assigned eight clusters to various interventions: improved water (W), improved sanitation (S), improved handwashing (H), improved nutrition (N), combined WSH, combined WSH + N, and a double-sized control arm. This current study kept the random allocation of clusters to intervention and control arms. We included four clusters within each block: 2 controls, 1 WSH, and 1 WSH + N. We combined the combined WSH and combined WSH + N into one intervention arm. We then compared the combined WSH (combined WSH and combined WSH + N) clusters and the control clusters.

Reporting for specific materials, systems and methods

We require information from authors about some types of materials, experimental systems and methods used in many studies. Here, indicate whether each material, system or method listed is relevant to your study. If you are not sure if a list item applies to your research, read the appropriate section before selecting a response.

Materials & experimental systems

n/a	Involved in the study	
☒	☐	Antibodies
☒	☐	Eukaryotic cell lines
☒	☐	Palaeontology and archaeology
☒	☐	Animals and other organisms
☐	☒	Clinical data
☒	☐	Dual use research of concern
☒	☐	Plants

Methods

n/a	Involved in the study	
☒	☐	ChIP-seq
☒	☐	Flow cytometry
☒	☐	MRI-based neuroimaging

Clinical data

Policy information about clinical studies

All manuscripts should comply with the ICMJE guidelines for publication of clinical research and a completed CONSORT checklist must be included with all submissions.

Clinical trial registration	NCT01590095
Study protocol	The protocol of the original study is available in the Open Science Framework (https://osf.io/tprw2/).
Data collection	From May 31, 2012, to July 7, 2013, 720 clusters were randomly allocated and 5551 pregnant women in 5551 compounds were enrolled to seven arms. Outcomes were measured after 12 months and 24 months (2014 and 2015, respectively) after the interventions were implemented. The trial was conducted in rural communities in Gazipur, Kishoreganj, Mymesingh, and Tangail districts. During community-based surveys, pregnant women were identified and enrolled, provided they were expected to give birth within the following 6 months. Caregiver-reported diarrhea was evaluated for children under 3 years of age at the time of enrollment and living within the compound. This study was conducted with informed consent from all participants. Data was gathered directly on handheld tablets through a custom study application developed using the Open Data Kit (ODK). The present analysis focused on index children within the birth cohort, and younger children residing in the same compound who were under the age of 3 years when the study started. Children with missing outcome data were omitted from the analysis. Our analysis only included observations during survey rounds 1 and 2 (2014 and 2015, respectively).

Outcomes

Primary and secondary outcomes were pre-specified in the protocol of the original study (https://osf.io/tprw2/). The primary outcomes included length-for-age Z-scores (LAZ) measured 24 months post-intervention implementation in index children and diarrhea prevalence in index and compound children under 3 years old at enrollment. Diarrhea was assessed at baseline among children under 3 years old and again 12 and 24 months post-intervention, using a definition of ≥3 loose or watery stools in 24 hours or ≥1 stool with blood, based on caregiver-reported symptoms with a 7-day recall period. Secondary outcomes included two additional measures of linear growth, child development metrics, and assessments of environmental enteropathy. In this pre-specified secondary analysis, we only focused on the diarrhea outcome, which is one of the primary outcomes. This variable was binary, representing '0' for the absence of the event and '1' for its occurrence.
